# Differentiated carbon reduction effects of clean heating policies: evidence from pilot projects in Northern China

**DOI:** 10.1186/s13021-026-00405-9

**Published:** 2026-02-07

**Authors:** Hongjie Ji, Handi Yang, Jintao Lu

**Affiliations:** 1 School of Economics and Management, Shanxi University, Taiyuan, 030006 China; 2 Sociology, St. Mary’s College, Durham University, Durham, UK; 3 School of Economics and Management, Taiyuan University of Science and Technology, No. 66 South Middle Ring Street, Taiyuan, 030024 China; 4 School of Economics and Management, Shanxi University of Electronic Science and Technology, Linfen, 041000 China

**Keywords:** Clean heating, Carbon emission reduction, Multiplier effect, Structural effect, Porter effect

## Abstract

As a far-reaching initiative in China’s air pollution control and energy transition efforts, the clean heating policy has sparked considerable debate in both academia and practice regarding its effectiveness in reducing carbon emissions. This study uses panel data from 15 prefecture-level cities in northern China from 2013 to 2023 and constructs a multi-period difference-in-differences model to empirically examine the impact of the clean heating policy on regional carbon emissions. The results are summarized as follows: (1) The policy effectively promotes the reduction of regional unit GDP and per capita carbon emission intensity in Northern China, but it has no evident effect on regional total carbon emissions. (2) The policy can exert the multiplier effect of the central government funds and structural effect to facilitate regional low-carbon transformation, but no significant Porter effect has been observed. (3) The carbon reduction effects exhibit significant regional heterogeneity. The policy has a more significant effect on carbon emissions of nonprovincial capital cities, coal-resource cities, and regions without coal power output, but it may significantly increase emissions in coal power-exporting regions. The clean heating policy should continue to be vigorously implemented, but its implementation strategy should be optimized by strengthening the transmission mechanism and addressing regional differences.

## Introduction

Winter heating is a rigid necessity for human survival in winter in cold regions, and the evolution of its energy supply methods profoundly affects regional environmental quality and carbon emission pathways [1, 2]. For a long time, the burning of scattered coal, as the primary heating method in rural areas and peri-urban regions in northern China, has not only provided heat but also caused severe air pollution, posing significant public health risks. It has become one of the main contributors to frequent smog occurrences [3–5].In response to this urgent environmental and public health challenge, the Chinese government has systematically promoted the Winter Clean Heating Policy in northern China since 2017, under the framework of the “*Northern Region Winter Clean Heating Plan (2017–2021)*”. Between 2017 and 2019, three batches of pilot cities were selected, totaling 43 cities, which received financial support from both central and local governments. The core of the clean heating policy is to replace traditional coal-burning heating methods through technical pathways such as “coal-to-electricity” “coal-to-gas”, and the use of renewable energy [6, 7].By the end of 2024, the policy is expected to cover around 70 million households, making it a widely influential and far-reaching systemic issue. It has achieved recognized success in reducing the concentration of conventional pollutants like PM2.5 and improving air quality [8, 9].However, after China set its strategic goals of “peaking carbon emissions by 2030 and achieving carbon neutrality by 2060” in 2020, the policy, which originally aimed at “pollution reduction,” now faces the pressing question of its “carbon reduction” co-benefits. Therefore, scientifically and accurately assessing the carbon emission reduction effects of the clean heating policy is crucial, not only for understanding the sustainability and optimization of this policy under the new “dual carbon” goals but also for advancing the understanding of the co-benefits of environmental regulation policies. It holds significant theoretical and practical importance for promoting the synergistic reduction of pollution and carbon emissions.

The current research on clean heating mainly includes three aspects. The first is the application and optimization of clean heating renovation technology, including the application of energy Internet [10] and the “dual simultaneous” renovation of building insulation and clean heating equipment [11]. The second is the study on the participants and modes in clean heating renovation, including the government’s leading role in government-led function [12], villagers’ willingness to participate [13], and multiparty collaborative adaptive mode [14, 15]. The third is the effect evaluation of the clean heating policy, which covers eco-environmental effect, economic effect [16, 17], and health and social effects [18]. The study on policy effect evaluation focuses on eco-environmental effect, and the investigation on eco-environmental effect concentrates on pollution reduction, that is, improving air quality. Scholars have continuously enriched and improved the index measurement and sample selection by engineering the calculation of coal substitution [19], metrology, and real-time monitoring [20], which confirmed the effectiveness of clean heating in reducing various air pollutants.

Despite a consensus on the air quality improvement effect of the clean heating policy in the academic circles, its influence on carbon emissions has not been sufficiently explored. Some scholars believe that the per capita carbon emissions of household heating energy are decreasing in the transformation to modern energy, but the decline rate is slow [21, 22]; the carbon emissions of different clean heating technology paths are quite different. The three paths contributing to the lowest carbon emissions are biomass particles, air source heat pumps, and air conditioners. Forecasts reveal that “by 2030, the annual carbon dioxide emissions of China will be reduced by 260 million tons based on the level in 2020” [23]. However, some scholars have reached the opposite conclusion. Based on the cases of different provinces in China, they have found that large-scale implementation of clean heating has an evident pollutant emission reduction effect, but it will lead to a sharp increase in CO_2_ emissions, which is not conducive to achieving carbon peaking and carbon neutrality goals [9, 24, 25]. This finding can be attributed to the carbon transfer caused by the increase in power demand and the increasing heating demand induced by urbanization. Some scholars have calculated the carbon footprint of a specific clean heating mode (coal to gas, coal to electricity, clean coal, photovoltaic power generation, wind power generation, hydropower generation, biomass, and ground source heat pump), compared it with the scattered coal mode, and reached differential conclusions [26–29]. They have focused on single heating modes, and the influence on regional overall carbon emissions remains unknown.

Current research on the clean heating policy has several limitations. Most studies focus on the effect evaluation of this policy on air quality improvement, but minimal attention has been paid to its carbon emission impact. The existing research on the carbon emission impact often stays at the level of descriptive, simple comparative analyses, and the policy transmission mechanism regarding the influence of the clean heating policy on the connotation of carbon emissions and the interregional differences in policy effects caused by resource endowments and economic and social factors have not been deeply explored. With the deepening of China’s “carbon peaking and carbon neutrality strategy,” whether the clean heating policy really reduces carbon emissions has been controversial in reality. In the annual target responsibility assessment of provincial governments by the central government of China, the carbon emission reduction of clean heating is estimated based on the amount of scattered coal replaced in terminal households, which is not scientific enough. Clean heating refers to a low-carbon-emission, low-energy-consumption heating mode through an efficient energy utilization system using natural gas, electricity, geothermal energy, biomass, solar energy, industrial waste heat, clean coal (ultralow emission), nuclear energy, and other clean energy sources, which includes the whole heating with the goal of reducing pollutant emission and energy consumption, involving clean heat sources, efficient transmission and distribution networks (heating networks), and energy-saving buildings (heat users). For example, the original scattered coal used for household heating is replaced by natural gas, electricity, biomass, and clean coal, among which natural gas and clean coal will still produce carbon emissions, and the carbon emissions of electricity will be transferred to the upstream power production link.

Hence, the following research questions are raised in this paper: Can the clean heating policy reduce carbon emissions? What transmission mechanism affects carbon emissions? Does spatial heterogeneity exist in the carbon emission impact? This paper aims to understand the carbon emission reduction effect of the policy deeply and provide support and guidance for policy optimization and implementation. The marginal contributions of this paper are as follows: First, the effect of clean heating on regional carbon emissions is quantitatively evaluated, the research on policy effects is enriched, and the debate between “whether the policy can reduce carbon emissions” and “whether to continue to promote the policy” is solved. Second, the transmission mechanisms of government financial funds’ multiplier effect, energy and industrial structure effect, and Porter effect are clarified, and theoretical, realistic guidance is provided for policy-based carbon emission reduction. Third, the regional heterogeneity in carbon reduction effect of the policy is analyzed from the aspects of urban administrative level, resource attributes, and presence/absence of coal power output to provide reference support for the implementation of regional differential policies.

The marginal contributions of this paper are as follows: First, it provides a quantitative assessment of the impact of clean heating policies on regional carbon emissions, thereby enriching the literature on policy evaluation and offering empirical evidence to inform the debate regarding the policy’s carbon reduction efficacy and its future implementation. Second, it elucidates the transmission mechanisms, including the multiplier effect of fiscal funds, structural effects in energy and industry, and the Porter effect, thereby furnishing a theoretical and empirical basis for refining emission reduction strategies. Third, it examines the regional heterogeneity of the policy’s effects based on city administrative level, resource endowment, and role in coal-power transmission, which can inform the design of spatially differentiated policies.

The remaining sections of this paper are structured as follows: “Theoretical analysis and research hypotheses”section presents four hypotheses based on theoretical analysis; “Econometric model” section introduces a difference-in-differences model to analyze the relevant data; “Benchmark regression and data analysis” section conducts baseline regression according to the model and variables, and verifies the robustness of the results through methods such as parallel trends, robustness checks, and heterogeneity analyses;“Conclusion and suggestions” section concludes the study and provides relevant policy implications.

## Theoretical analysis and research hypotheses

This paper introduces the theory of environmental externalities and the theory of public goods to construct the analytical framework. The former provides a key perspective and states that the nonmarketization influence of economic subject activities on the environment is not fully reflected in economic transactions, which results in the distorted allocation of market resources [30]. Traditional heating methods, for example, scattered coal combustion, have significant negative externalities [31], and their carbon emissions affect climate [32], environment, and human health, but the cost is not entirely borne by users [33]. The clean heating policy can correct externalities, optimize the energy structure, and reduce carbon emissions by means of subsidies and supervision. However, its impact on carbon emissions cannot be comprehensively understood only by correcting market distortions, and theory on public goods deepens the analysis on this basis. This theory holds that the market supply of noncompetitive, nonexclusive public goods will fail easily. The results of carbon emission reduction brought by clean heating are public goods, and individuals lack incentives to promote clean heating to achieve carbon emission reduction, so government intervention is required. The government implements the clean heating policy, invests public resources to compensate for market failures, and popularizes clean heating to curb carbon emissions. This theory follows theory of environmental externalities from the necessity of government intervention and explains the rationality and importance of government policy regulation. The two theories echo each other. Theory of environmental externalities reveals market defects, and theory of public goods clarifies the basis of government intervention, which together form a complete theoretical framework.

### Carbon emission reduction effect of clean heating

The clean heating policy copes with the above problems by economic and supervision means. Subsidies are provided for infrastructure construction, household heating equipment, and energy, which reduce the cost of clean heating for households and guide consumers to consider environmental costs. However, strict standards and regulatory measures should be formulated to limit the use of scattered coal, punish violations, and promote the internalization of externalities. Clean heating renovation has been performed in various ways in Northern China. Centralized heating and electric heating terminals do not directly produce carbon emissions; natural gas heating is more environmentally friendly because the carbon emissions per unit of heat from natural gas are lower than those from scattered coal. In renewable energy heating, the carbon emission induced by biomass burning is lower than that of scattered coal burning, accompanied by carbon neutrality, and carbon emissions are not directly produced by heating with other renewable energy. Moreover, the household renovation equipment for clean heating has been purchased by the government and achieved higher energy efficiency than traditional stoves. As the proportion of clean energy in the heating field increases, direct carbon emissions from scattered coal combustion are significantly reduced.

The carbon emission reductions brought about by clean heating have the characteristics of a public good, and individuals can benefit from the overall environmental improvement, whether they participate in the transformation or not [34] (Haines et al., 2007). Given the nature of public goods, however, private sectors lack incentives to provide adequate clean heating services, and individual decision making ignores social benefits; consequently, the supply of clean heating is below the optimal level and fails to reduce carbon emissions fully. To correct market inefficiencies, the government invests public resources in implementing clean heating policies. Fiscal subsidies and dedicated funds are provided to reduce the costs borne by households and enterprises and to enhance the accessibility of clean heating. Moreover, industrial policies are designed to foster the growth of related sectors, stimulate research, development, and deployment of clean energy technologies, and facilitate market formation. In turn, the advancement of clean heating further motivates upstream enterprises to optimize energy-saving technologies and drives innovation among equipment manufacturers, which lead to reductions in indirect carbon emissions [35].

### Mediating role played by the multiplier effect of central government’s financial funds

According to theory of environmental externalities, the clean heating policy aims to correct the traditional heating externalities and promote the internalization of environmental costs. The central government, as the initiator and promoter, issued the *Winter Clean Heating Plan for Northern China* (2017–2021) that requires governments at all levels to set up a dedicated working group, build a coordination mechanism, and strengthen policy supervision and evaluation, and the central government’s financial subsidy plays a key role. Subsidies reduce the relative cost of clean heating [36], which encourages households to adopt clean heating options, decrease the use of high-carbon heating methods, and provide an initial correction of the negative externalities associated with traditional heating. Subsidies have a “multiplier effect.” With its authority and regulatory power, the central government stimulates local financial input and enterprise investment through financial subsidies and awards [37].

Based on the theory of public goods, local governments, driven by central fiscal support, establish dedicated financial subsidy management systems to subsidize energy prices and terminal equipment, while increasing investments in urban infrastructure construction, such as laying natural gas pipelines and upgrading power grid infrastructure. These measures guide residents toward clean heating, thereby promoting the effective provision of carbon reduction as a public good. These actions have promoted the construction of a clean energy consumption structure, further reduced carbon emissions, and more deeply and comprehensively corrected externalities. Official statistics from 2017 to 2019 show that the central government invested 49.3 billion yuan and pushed local financial input to be tripled, which revealed a marked “multiplier effect”.

### Mediating role played by energy and industrial structure effect

The energy structure is influenced by the clean heating policy in multiple aspects. On the one hand, the clean heating policy reduces the demand for scattered coal in Northern China, guides the establishment of a cleaner energy consumption structure, diminishes the total amount and intensity of energy consumption by utilizing efficient equipment, realizes the low carbon emission of the energy consumption structure, and directly corrects the negative externalities of scattered coal heating. Enormous investment and scientific planning are needed in early-stage clean heating renovation, but enterprises and households, which pursue their own benefits and consider risks, are not willing to take an active part. In this case, according to theory of public goods, policy guidance and support compensate for the market failure and promote the effective supply of carbon emission reduction as a public good [38]. Moreover, the policy drives the upstream supply of cleaner energy, enhances the utilization of renewable energy, and fosters a low-carbon transformation of the energy production structure.

The clean heating policy has also profoundly changed the relevant industrial structure, especially in the equipment manufacturing industry and service industry, and makes important indirect contributions to regional carbon emission reduction [39]. Specifically, this policy promotes the upgrading of the equipment manufacturing industry to reduce carbon emissions indirectly. Market demands urge enterprises to increase R&D, develop efficient, environmentally friendly heating equipment, and promote the upgrading of the manufacturing industry. This approach, in turn, drives upgrades in the manufacturing sector while improving production processes, enhancing automation levels, and reducing energy consumption and emissions [40]. Moreover, industrial development stimulates the green transformation of supply chains, and upstream suppliers adopt environmentally friendly materials and processes. This approach drives the greening of the manufacturing sector, reducing carbon emissions, and mitigating the negative externalities of high-carbon emissions in traditional equipment manufacturing. The popularization of clean heating equipment has greatly increased the demand for installation and maintenance services, and professional service companies have developed to improve energy efficiency and reduce emissions by optimizing equipment operation, and create jobs. The market for energy management and technical consulting services is also expanding due to demand. Professional companies customize schemes for users by virtue of technologies, which optimizes energy use and reduces emissions. Based on theory of public goods, despite the effect of such service sectors in energy efficiency improvement and emission reduction, single enterprises or individuals lack momentum due to the input–output ratio and technology thresholds. Conditions are created by the policy implementation for such enterprises or individuals, which facilitates the supply of carbon emission reduction as a public good.

### Mediating role of Porter effect

The “Porter effect” means that properly designed environmental regulation policies can stimulate the compensation effect of enterprise innovation and achieve a win-win situation for the economy and environment. From the micro level of enterprises, the clean heating policy brings energy saving and emission reduction pressure to equipment manufacturers similar to a “baton” of environmental regulation and stimulates the “Porter effect” [41]. For survival and development, enterprises invest resources in technological innovation based on the influence of theory of environmental externalities and develop efficient low-carbon heating equipment [42]. Their innovation products not only meet clean heating demands, reduce carbon emissions, and correct the negative externalities of traditional heating but also expand the market and improve economic benefits by technology advantages, transform external costs into competitive edges, and realize a win-win pattern. This win-win pattern facilitates enterprises to lay a greater emphasis on green technology R&D and continuously reduce carbon emissions. The clean heating policy stimulates enterprise innovation by pressuring them, influences their production and management, and helps them develop efficient products and increase benefits.

In the field of energy supply sector, the clean heating policy promotes the clean transformation of energy production and supply companies as pointed out by theory of external externalities. Under policy guidance, traditional coal enterprises increase their R&D input into clean coal utilization technologies or transform into the development of clean energy business to avoid the negative externalities of high carbon emissions. Through technological innovation and business transformation, enterprises reduce their own carbon emissions, correct the externalities of production links, provide clean energy products to facilitate carbon emission reduction, and carve new paths to enhance competitiveness and sustainable development capability. The clean heating policy promotes the transformation and innovation of energy enterprises and achieves multiple goals, such as carbon emission reduction and competitiveness improvement.

From the macro industrial level, the clean heating policy triggers the Porter effect, drives the technological innovation and upgrading of the industrial chain, and compensates for the market failure of carbon emission reduction as a public good. In terms of clean heating equipment manufacturing, upstream and downstream enterprises innovate to adapt to the policy and market demands, for example, part suppliers develop energy-saving components, and material suppliers provide efficient thermal insulation materials [43]. The collaborative innovation of industrial chains improves industrial production efficiency and technological level and drives the green, low-carbon optimization of the industrial structure. This approach strengthens the carbon emission reduction effect and realizes the effective supply of carbon emission reduction as a public good. Driven by innovation, industries achieve a higher level of sustainable development and economic–environmental coordination. This process embodies that industrial behaviors are guided by the policy and contribute to carbon emission reduction and sustainable industrial development.

Based on the above analysis, the following hypotheses are proposed:

#### Hypothesis 1

(H1): The clean heating policy has a significant inhibitory effect on regional carbon emissions.

#### Hypothesis 2

(H2): The multiplier effect of the central government’s financial funds mediates the clean heating policy and the carbon emission reduction effect.

#### Hypothesis 3

(H3): Energy and industrial structure mediates the clean heating policy and the carbon emission reduction effect.

#### Hypothesis 4

(H4): The Porter effect mediates the clean heating policy and the carbon emission reduction effect.

**Fig. 1:**
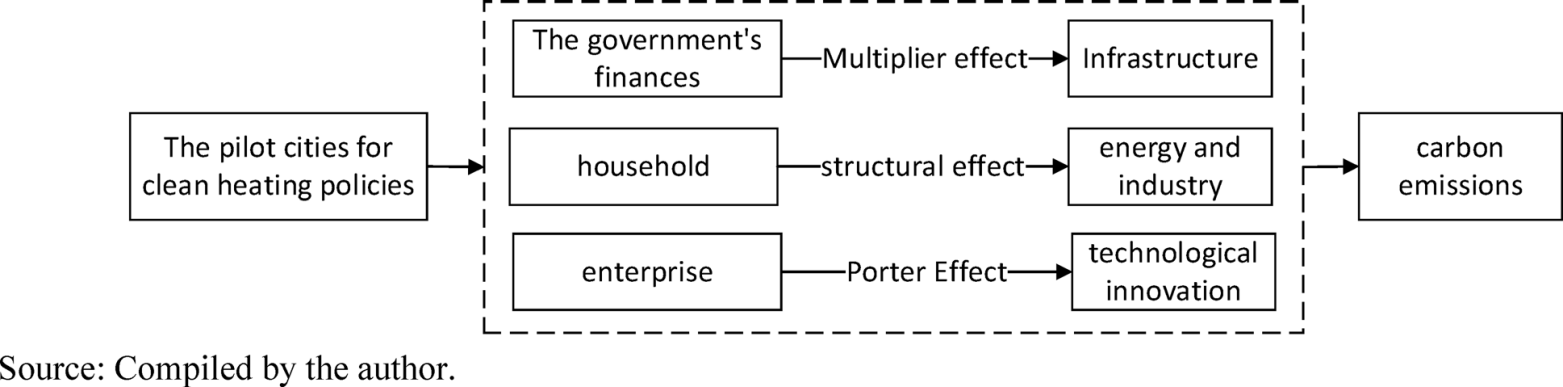
Theoretical analysis framework

The theoretical model of this paper is shown in Fig. 1.

## Econometric model

### Modeling

#### Multistage difference-in-difference model

In this paper, the clean heating pilot policy was regarded as a “quasi-natural experiment.” The difference-in-difference (DID) model could effectively control the interference of time trend on the policy evaluation effect, and the individual fixed effect was introduced to control the inherent differences in different regions, so that the evaluation results could better reflect the changes brought about by policy implementation, rather than the results caused by interregional differences. The traditional DID is only suitable for evaluating the policy at a single point, while the clean heating policy pilot was gradually promoted in different years, and different batches of the policy were treated at different times. Therefore, the practice of relevant literature [43] (Liu & Xu, 2024) was referenced to establish the regression equation of the clean heating policy pilot for carbon emission reduction based on the multistage DID model design, and then the practical effect of the policy pilot was evaluated using the high-dimensional fixed effect regression.

1 $$\:{TC}_{it}={a}_{0}+{a}_{1}{treat}_{it}+{a}_{2}{X}_{it}+{u}_{i}+{\lambda}_{t}+{\epsilon}_{it}$$

2 $$\:{CIgdp}_{it}={a}_{0}^{{\prime\:}}+{a}_{1}^{{\prime\:}}{treat}_{it}+{a}_{2}^{{\prime\:}}{X}_{it}+{u}_{i}+{\lambda}_{t}+{\epsilon}_{it}^{{\prime\:}}$$

where $\:i$ and $\:t$ are the prefecture-level city and year, respectively; $\:TC$ and $\:CIgdp$ are the explained variables representing the regional total carbon emission and the carbon emission intensity per unit GDP, respectively; X represents a group of control variables significantly influencing the carbon emission; $\:{treat}_{it}$ indicates whether a prefecture-level city enters the scope of the central financial subsidies for the clean heating pilot, which is defined as 1 for the treatment group and 0 for other regions in year $\:t$, and its coefficient $\:{\alpha\:}_{1}$ denotes the net influence of heating renovation on carbon, which is the emphasis of this paper. The policy treatment effect reflects the influence of the policy on the carbon emission intensity in the treatment group and control group. According to the theoretical analysis results, this coefficient should be negative, that is, after policy implementation, total carbon emissions and carbon intensity in cities with the heating system show a marked declining trend. In this paper, the individual fixed effect $\:{u}_{i}$ and practical fixed effect $\:{\lambda\:}_{t}$ were controlled, and the standard error $\:{\epsilon}_{it}$ was clustered to the urban level. The descriptive statistical results of variables are listed in Table 1, and the standard error of urban carbon emission intensities is large, which indicates the great interregional difference in carbon emission.

**Table 1:** Descriptive statistics of main variables

Variability		Sample	Mean	Standard	Minimum	Maximal
Total carbon emissions (CEADs)	TC1	1397	24.8302	43.4223	3.8366	498.7561
Total carbon emissions (EDGAR)	TC2	1397	40.4599	30.7007	3.2737	194.2765
Carbon emissions per unit of GDP (CEADs)	CIgdp1a	1,397	0.2507	0.1448	0.0198	1.1289
Carbon emissions per unit of GDP (EDGAR)	CIgdp1b	1,397	7.4926	1.1186	4.6347	11.7962
Carbon emissions per capita (CEADs)	CIpop2a	1,397	0.1265	0.1360	0.0130	2.6555
Carbon emissions per capita (EDGAR)	CIpop2b	1,397	6.7102	1.2179	3.9831	11.2492
Investment in municipal public facilities	llf	1397	594,172	382,018	310	1.37 × 10^7^
Clean energy structure	ces	1397	0.1454	0.1401	0.0029	1.7318
Upgrading of an industrial structure	iup	1397	2.3069	0.1472	1.8312	2.8357
The Porter Effect	lnginv	1397	2.4781	1.8188	0.0000	9.2113
Population size, population	lnpop	1,397	5.7260	0.7646	3.1781	7.2538
Commercial scale	iad	1,397	1.1183	0.6603	0.1136	5.2968
per capita GDP	pcgdp	1,397	0.1679	0.1967	0.0095	2.0639
Number of patents	lnogp	1,397	4.5490	1.6573	0.0000	10.0112
Mean temperature of air	at	1397	124.2360	78.8651	−16.8432	272.4673
mean wind speed, mean wind velocity	aws	1397	60.7761	77.9764	49.3632	85.3691

#### Mechanism test model

Given such controversies as endogeneity over the mediating effect test through the traditional “three-step” method, the practice of Jiang T [44] (2022) was referenced in this paper, and Eqs. (1) and (2) were combined to establish the “two-step method” to assess the mediating roles played by the “multiplier effect,” structural effect, and Porter effect and to investigate the influence of the clean heating policy on regional carbon emissions.

3 $$\:{M}_{it}={\beta\:}_{0}+{\beta\:}_{1}{treat}_{it}+\sum\:{\beta\:}_{2}{X}_{it}+{u}_{i}+{\lambda\:}_{t}+{\epsilon\:}_{it}$$

where M is the internal mechanism variables, namely, clean energy structure ($\:ces$) and industrial structure upgrading ($\:iup$).

### Variables and data interpretation

#### Explained variables: dual control over the amount and intensity of carbon emissions

The core explained variables in this paper were the dual control targets of carbon emissions, namely, total carbon emission ($\:TC$) and carbon emission intensity ($\:CIgdp$), which are generally considered the core indicators to measure the regional carbon emission level (Zhao & Mao, 2012) and important metrics for the central government’s assessment of provincial governments’ responsibilities in controlling greenhouse gas emissions. The logarithmic values of total carbon dioxide emissions and carbon dioxide emission intensity per unit GDP were used to measure carbon emissions and carbon intensity at the prefectural city level, respectively. A cross-validation was performed using the carbon emission data from the Carbon Emission Accounts and Datasets (CEADs) of China and the Emissions Database for Global Atmospheric Research (EDGAR) of the EU. On this basis, the carbon emission data of prefecture-level cities were subject to inversion using nighttime light data.

#### Explanatory variable: clean heating policy

**Fig. 2:**
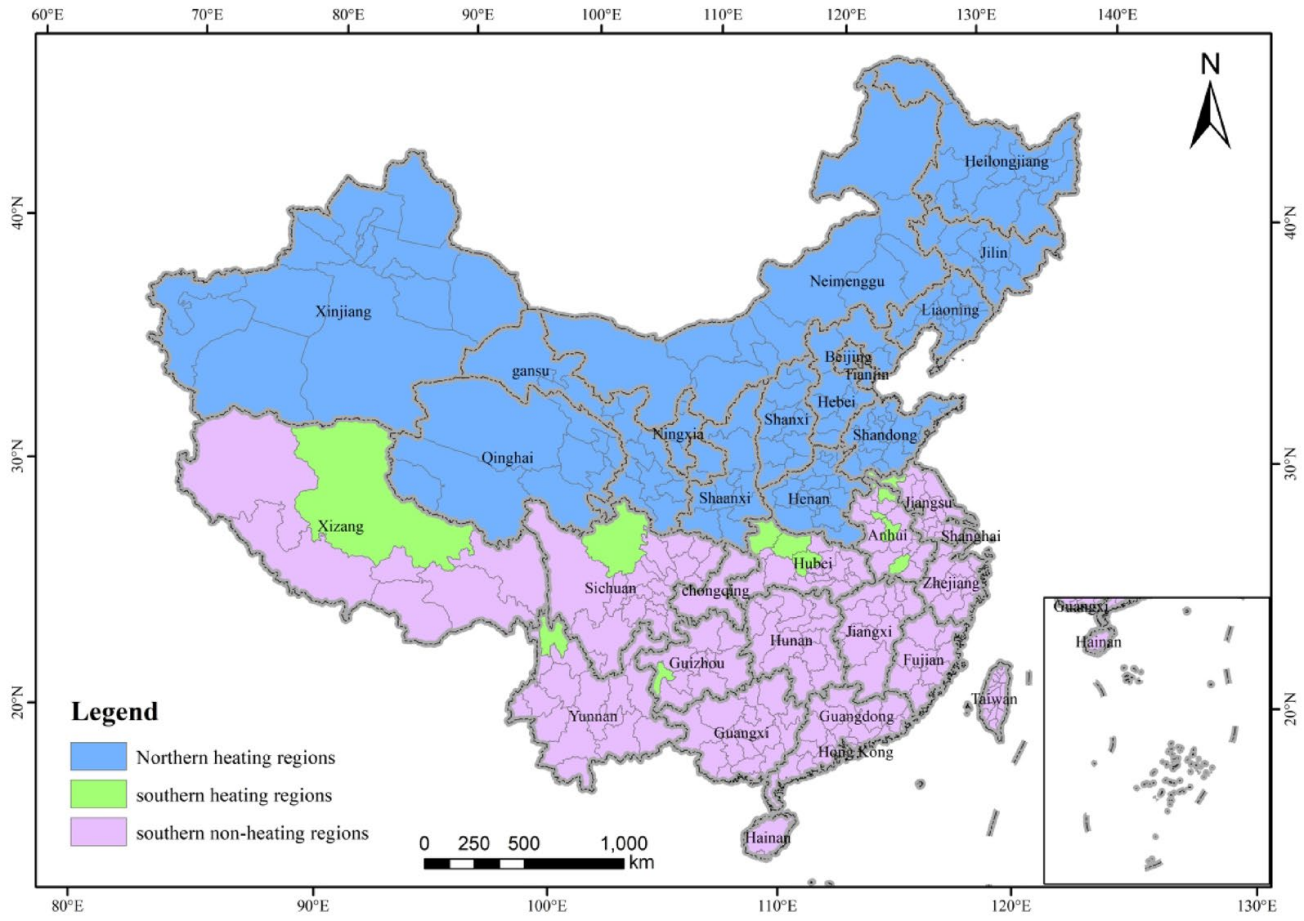
Regional division of heating zones in China

**Fig. 3:**
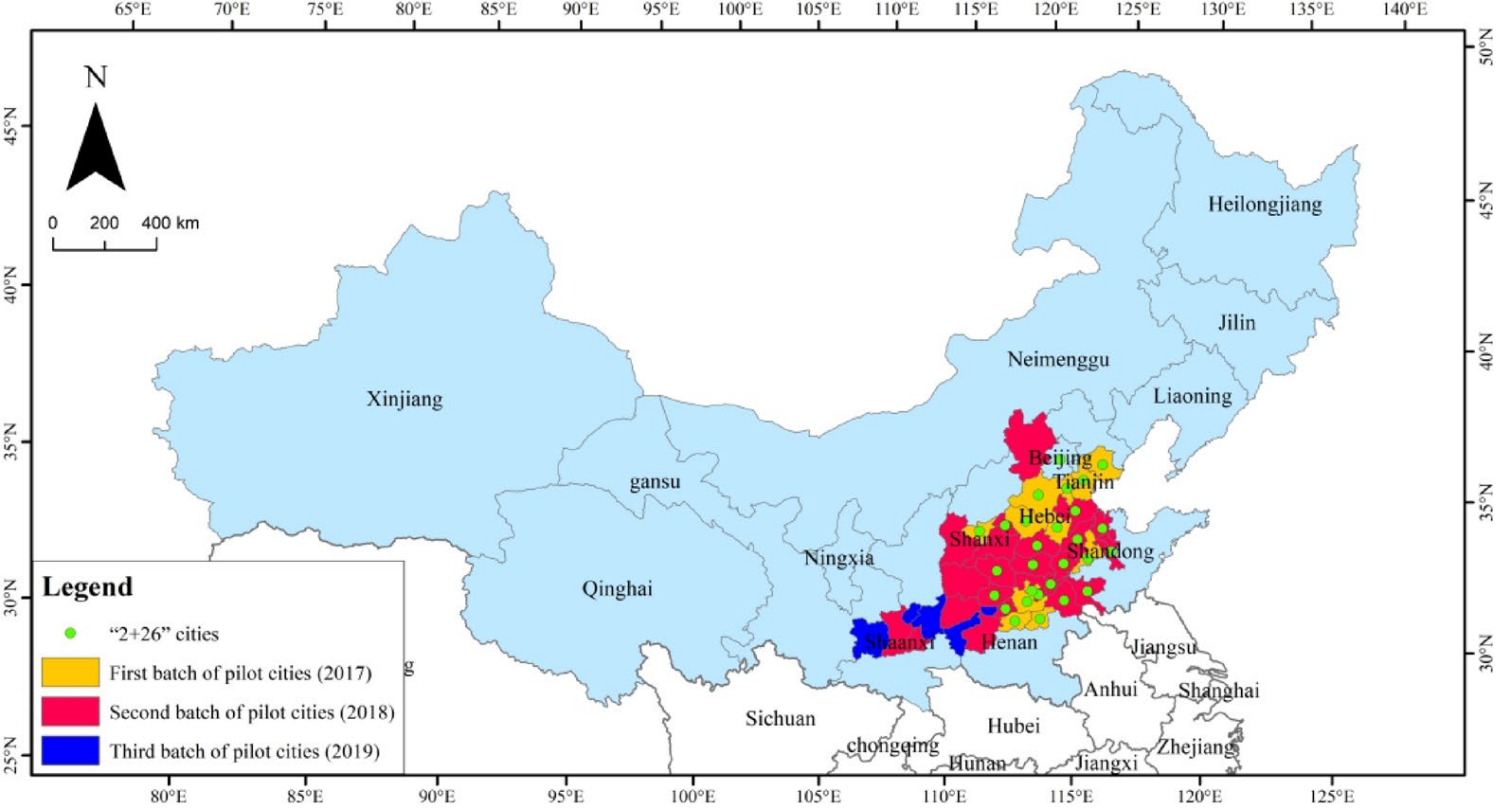
Phased distribution of pilot cities for clean heating policies

The core explanatory variable selected in this paper was the pilot city of the clean heating policy. Referring to Beck’s classic design, a city is 0 before inclusion in the pilot, and 1 in and after the year of inclusion in the policy pilot (Beck et al., 2010). The heating boundary line (the Qinling Mountains–Huaihe River Line) is widely recognized as the geographical dividing line between North and South China, with cities to the north of the boundary serving as the control group samples(Figs. 2 ).A total of 43 cities in 3 clean heating pilot batches were obtained as the samples in the treatment group according to relevant formal documents, such as the *Notice on Publicizing the List of Winter Clean Heating Pilot Cities in Northern China in 2017*, the *Announcement of the Second Batch of Central Financial Support for Winter Clean Heating Pilot Cities in Northern China in 2018*, and the *Notice on Issuing the Budget for Air Pollution Prevention and Control in 2019 *( Table 7 and Fig. 3).

#### Mechanism variables

Leverage of local financial funds ($\:llf$). The “multiplier effect” of the central financial input in recent years on local financial funds is restricted by the unavailability of accurate clean heating data, and the effect itself is also dynamic. In this paper, the investment in urban public facilities construction was chosen as the proxy variables for the local government input, with indicators derived from the sum of “energy conservation and environmental protection” and “urban and rural communities” in the general public budget. The data is sourced from the statistical yearbooks of various cities.

Clean energy structure ($\:ces$). In this paper, the clean energy structure index of prefecture-level cities was established through the following method: First, according to the index setting by Tang X F et al. (2011) over low-carbon energy consumption, the indicators used to characterize the clean energy consumption structure were set as the proportion of power, natural gas, and new energy consumptions in the total energy consumption. Second, the clean energy production structure index was set as the proportion of non-coal energy in primary energy production. Finally, the clean energy consumption structure index and the clean energy production structure index were averaged to acquire the clean energy structure index of a region. The energy consumption data mentioned above is sourced from the statistical yearbooks of various cities.

Industrial structure upgrading ($\:iup$). Referring to the research, different weights (0.682 and 0.318, respectively) were assigned to the rationalization of the industrial structure and the advancement of the industrial structure, and the comprehensive index of industrial structure upgrading was calculated [45]. The value-added data for the above industries is sourced from the statistical yearbooks of various cities.

Porter effect ($\:lnginv$). According to the research conducted by Zhang Q H and Bian Z Q [46], the Porter effect was characterized using the number of inventions and the number of green invents acquired by a city in the current year, respectively. The above data is sourced from the EPS database.

#### Control variables

To ensure the accuracy of the model estimation and effectively mitigate the endogeneity issue caused by omitted variables, thereby more clearly identifying the net effect of clean heating policies on regional carbon emissions, this paper selects a series of control variables based on classical environmental economics theory and related literature, considering four dimensions: economic development, urban development, population size, and natural meteorological conditions. The economic development variables are used to control the fundamental impact of economic scale and structure on carbon emissions, including indicators such as per capita GDP (pcgdp)and industrial scale(iad). The urban development and technological dimension variables are used to control the potential effects of urban modernization levels and technological innovation capacity on energy greening, such as population size (lnpop). The meteorological conditions variables are used to control the short-term fluctuations in energy demand (especially heating demand) and atmospheric diffusion conditions due to natural factors, including average temperature (at) and average wind speed (aws). The above economic development, urban development, and population data were derived from such statistical yearbooks as *China City Statistical Yearbook* and *China Urban Construction Statistical Yearbook*, and meteorological data came from “China daily surface climate dataset” established by the National Meteorological Data Center of China Meteorological Administration. Based on the data from each meteorological station, the daily meteorological data of each city were calculated through the weighted average method, the missing urban meteorological data were subject to neighborhood data supplementation, and the mean values of annual meteorological data were obtained according to daily data.

##  Empirical Results and Analysis

### Benchmark regression analysis

The multistage DID model under the double fixed effect was used to verify the impact of the clean heating policy on carbon emissions. Table 2 reports the regression results regarding the effect of the clean heating pilot on regional carbon emissions. TC1 and CIgdp1 are the total carbon emission and carbon emission intensity per unit GDP of a prefecture-level city obtained through nighttime light data inversion based on the provincial carbon emission lists in CEADs, respectively. TC2 and CIgdp2 are the total carbon emission and carbon emission intensity per unit GDP of a prefecture-level city based on EDGAR, respectively. In the regression analysis displayed in Columns (1), (3), (5), and (7), control variables are not added to investigate the basic relationship between the clean heating policy and regional carbon emissions; in Columns (2), (4), (6), and (8), the regression results after adding control variables are reported to analyze the actual emission reduction effect of this policy in complex realistic environments more comprehensively and precisely. The estimation coefficient of the clean heating policy is significantly negative for the carbon emission intensity per unit GDP at 1% level but irrelevant to the total carbon emission; hence, the clean heating policy plays a significant role in promoting the decline of regional carbon emission intensity in prefecture-level cities but has no significant effect on the regional total carbon emission. In addition, the regression results of control variables are consistent with expectations, especially the estimation coefficient of per capita GDP is significantly positive, that is, the increase in per capita GDP will increase the carbon emission intensity. This policy is uncorrelated with the regional total carbon emission, and the coefficient is positive.

**Table 2:** Baseline regression results

Variability	TC1		CIgdp1		TC2		CIgdp2	
	(1)	(2)	(3)	(4)	(5)	(6)	(7)	(8)
Clean heating points	0.8362 (0.8594)	0.6617 (0.8075)	−0.0305*** (0.0092)	−0.0246*** (0.0076)	1.3691 (1.5223)	0.9634 (1.5511)	−0.1593*** (0.0370)	−0.1124*** (0.0318)
Control changing	No control	No control	No control	control	No control	control	No control	control
Observations	1,397	1,397	1,397	1,397	1,397	1,397	1,397	1,397
City fixed effects	control	control	control	control	control	control	control	control
Time fixed effects	control	control	control	control	control	control	control	control
R^2^	0.9895	0.9897	0.8420	0.8476	0.9632	0.9609	0.9740	0.9961

Note: All regressions control for city and time fixed effects, with standard errors clustered at the city level. The coefficients in parentheses are reported as standard errors. *, **, *** indicate significance at the 10%,5%, and 1% levels, respectively. The same applies below

### Parallel trend test

To investigate the dynamic effect of the implementation of the clean heating pilot policy in Northern China, the practice of Kong et al. was referenced, and the event study method was adopted [47]. The specific model is displayed in the following formula:

4 $$\:{CIgdp}_{it}={\beta\:}_{0}+{\alpha\:}_{j}\sum_{j=-6}^{j=4}{treat}_{i,t+j}+{\beta\:}_{1}{X}_{it}+{u}_{i}+{\lambda\:}_{t}+{\epsilon\:}_{it}$$

where $\:{\alpha\:}_{j}$ is the estimation coefficient, which can reflect the difference in time trend between pilot regions and non-pilot regions; subscript $\:j$ denotes the interval of years since a prefecture-level city is included in the clean heating policy pilot in year $\:t$ until year $\:t+j$; $\:{treat}_{i,t+j}$ is 1 upon the $\:j$-th year after the prefecture-level city is selected into the pilot in year $\:j$, and 0 otherwise. Other variables are defined consistently with those in Eq. (1).

**Fig. 4:**
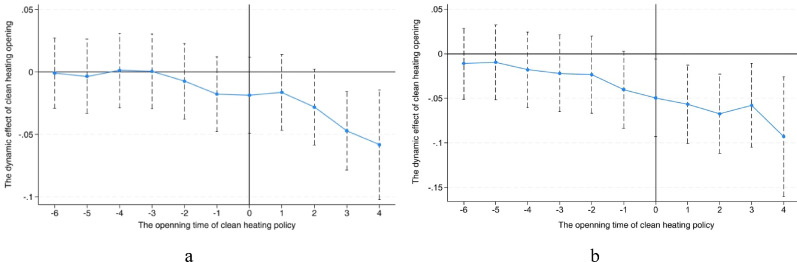
**a** Results of the Parallel Trend Test for CIgdp1. **b** Results of the Parallel Trend Test for CIgdp2

The parallel trend test results in Fig. 4 shows that before the implementation of the policy, the coefficient of the time variable was not significantly different from 0, that is, the variation trend of air quality in pilot cities and nonpilot cities was the same, without any policy effect; after the policy was implemented, the coefficient of the time variable was significantly different from 0, namely, since the year of policy implementation, the pilot policy already generated a marked effect, which coincided with the actual heating renovation situation, so the parallel trend test was passed.

### Robustness analysis

#### Placebo test

**Fig. 5:**
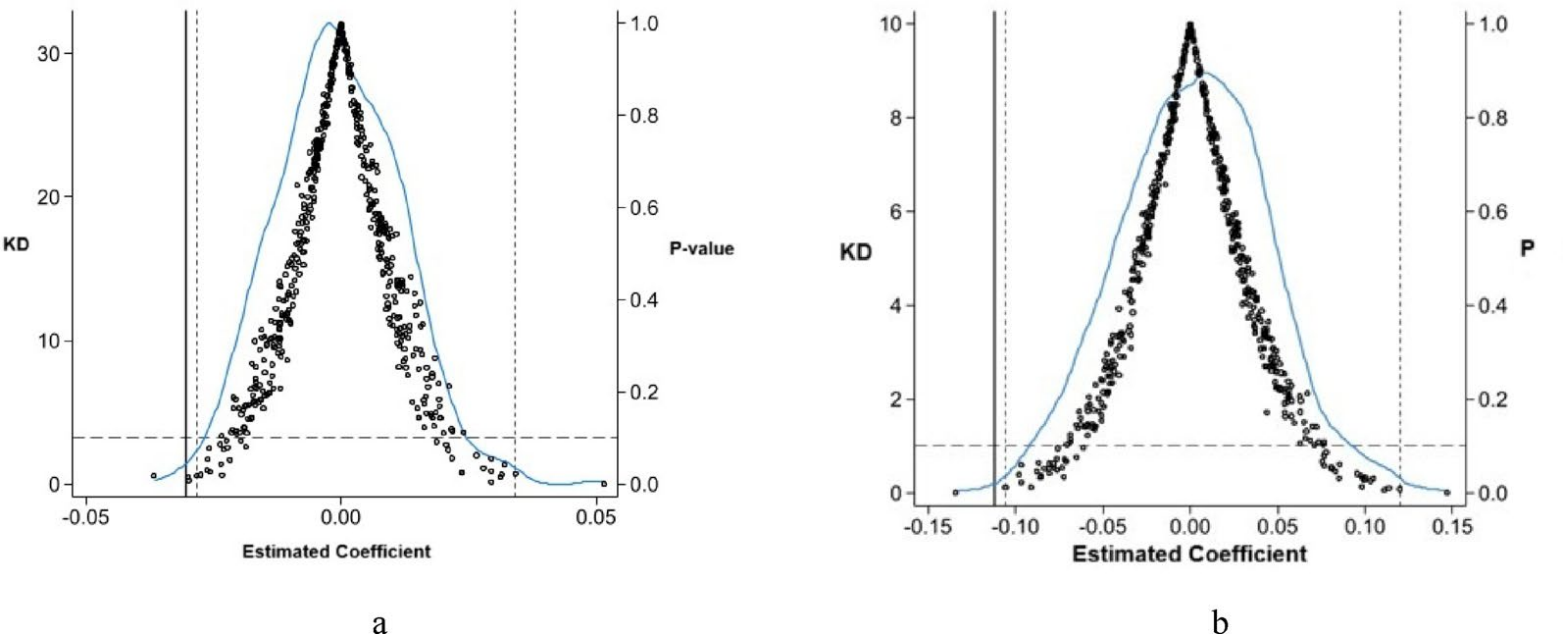
**a** Placebo test results for CIgdp1. **b** Placebo test results for CIgdp2

1. To verify that the impact of the clean heating policy on regional carbon emissions was not caused by random factors, a placebo test was further adopted to identify the contingency of the pilot effect. By reference to the practice of Ferrara et al. [48], random sampling was performed 500 times according to the implementation of policy implementation in the basic regression, a “pseudo-policy dummy variable” was established, a regression estimation was conducted once again as per Model (1), and the estimation coefficient and p value distribution were tested, as shown in Fig. 5. The results indicate that the estimates from the baseline regression are not biased due to potential omitted variables. Therefore, the conclusion that clean heating policies can significantly promoe regional carbon emission reductions is reliable.

#### Replacement of the explained variable

2. To prove further that the clean heating policy could reduce the carbon emission in Northern China, the explained variable measuring the regional carbon emission was replaced, and a robustness test was completed through the regression of the per capita carbon emission intensity in each prefecture-level city. The regression results in Table 8 reveal that after the explained variable was replaced, the regression results still prove that the clean heating policy exerts a good carbon emission reduction effect.

#### Sample selection bias

3. Because the designation of clean heating pilot cities considered factors such as the geographical location, resource endowments, and economic development level of cities, some cities had a greater probably of includsion in the pilot, which led to the biased error of sample selection in this paper. Two strategies were adopted to avoid the interference of the biased error with the research conclusions, improve the exogeneity of the pilot policy impact, and ensure the robustness of research conclusions.

First, the subsamples not including provincial capital cities were used for the analysis. Given the scale, economic level, and political advantages of provincial capital cities, they had increased probability to become pilot cities. Hence, provincial capital city samples were excluded. The regression results based on subsamples are listed in Column (1) of Table 9, and the correlation coefficient remained significantly negative, which manifest that after excluding provincial capital cities, the clean heating policy can reduce the regional carbon emission intensity.

Second, “2 + 26” cities were estimated. “2 + 26” cities refer to the cities in the Beijing–Tianjin–Hebei air pollution transmission channel, which presented the highest air pollution and emission intensity (emission per unit national territorial area) due to such factors as the coal-dominated energy structure, particular stress of the industrial structure on the chemical industry, and highway-dominated transportation network. Hence, an air pollution prevention and control leading group for Beijing, Tianjin, and Hebei and surrounding areas was founded in 2013, a series of environmental protection measures was introduced, and environmental supervision was strictly executed, which might promote carbon emission reduction. “2 + 26” city samples were excluded. The regression results based on subsamples are listed in Column (2) of Table 9, and the correlation coefficient was still markedly negative, which reflect that the “2 + 26” city samples have not generated any substantial influence on the estimation result.

#### Exclusion of influence of environmental policies in the same period

4. Environmental regulation has a synergistic effect on carbon emission reduction while mitigating air pollution. During the “Twelfth Five-Year Plan” and “Thirteenth Five-Year Plan” of this sample study, China issued multiple environmental policies, and the pilot areas of relevant policies overlapped, so the resulting pollution reduction may also reduce the regional carbon emission intensity. Therefore, in this paper, representative environmental policies in the research period were selected, including “low-carbon cities,” “carbon emission permit trading pilot,” and “ten articles for air pollution control,” which covered a wide range, and thus they were excluded based on the following method.

Exclusion of “low-carbon cities” policy. China’s “low-carbon cities” started in 2010 to promote ecological civilization and realize the goal of greenhouse gas emissions. Since the implementation, 81 national low-carbon city pilots were carried out in three batches successively. Referring to the practice of Xie Q et al. [49] (2023), the interaction term between the “low-carbon cities” policy and the dummy variable of year was added based on the basic model to control the influence of the policy of “low-carbon cities” on the carbon emission reduction effect of clean heating. The regression results in Table 10 reveal that the coefficient of the core explanatory variable remained significantly positive, which manifests that the basic regression conclusions remain robust.

Exclusion of “carbon emission permit trading pilot” and “ten articles for air pollution control.” China’s “carbon emission permit trading pilot” was initiated in 2011, and 8 pilot provinces or cities, namely, Beijing, Shanghai, Tianjin, Chongqing, Hubei, Guangdong, Shenzhen, and Fujian, were determined successively, among which Beijing and Tianjin are located in Northern China and belong to provincial capital cities. Hence, the effect of this policy was excluded in the same way as specified in the analysis results of “subsamples not including provincial capital cities.” The “ten articles for air pollution control” policy is namely the *Action Plan for Air Pollution Prevention and Control* introduced by the State Council in 2013, which lays a particular emphasis on Beijing–Tianjin–Hebei and surrounding areas, the Yangtze River Delta region, Fenhe–Weihe Plains, and Sichuan Province. Among them, Beijing–Tianjin–Hebei and surrounding areas and Fenhe–Weihe Plains in Northern China coincided with “2 + 26” cities, so the effect of this policy was excluded consistently with the above analysis result of exclusion of “2 + 26” cities.

### Mediating effect analysis

The above empirical results show that the implementation of the clean heating policy has significantly reduced the carbon emission intensity in Northern China. Thus, the influence path of this policy on the carbon emission intensity was further tested. To assess the mediating roles played by the multiplier effect of central government’s financial funds, structural effect, and Porter effect in the influence of the clean heating policy on the carbon emission in pilot regions, a mediating mechanism test was performed (Wen & Ye, 2010), and the results are listed in Table 3. Columns (1) and (2), Columns (3) and (4), Columns (5) and (6), and Columns (7) and (8) indicate the mediating roles played by the multiplier effect of central government’s financial funds ($\:llf$), the clean energy structure ($\:ces$), the industrial structure upgrading ($\:iup$), and the Porter effect ($\:lnginv$) in the carbon emission reduction effect of the clean heating policy, respectively.

The regression results (Table 3) reveal that the clean heating policy has boosted the scale of infrastructure investment in pilot cities to some extent; thus, H2 was verified, that is, the central government’s financial rewards and subsidies guide local governments to increase the investment in urban infrastructure construction, lay natural gas pipeline networks, and optimize power infrastructure. The clean heating policy had a significant positive influence on the cleaning of the regional energy structure, but the significance declined after control variables were added. The possible reason is that residential heating energy consumption and even residential life energy consumption account for a small proportion in the regional total energy consumption, with specific uncertainties; however, the cleaning of the regional energy structure will certainly reduce the regional carbon emission. The clean heating policy significantly positively affects the regional industrial structure upgrading. According to relevant research conclusions, the advancement and rationalization of the industrial structure produce a marked inhibitory effect on carbon emissions by improving the green total factor productivity, so H3 was verified. However, the clean heating policy was uncorrelated with regional green technological innovation, and H4 was not verified. Considering that it may be difficult to effectively capture the cross-regional flow characteristics of clean heating technologies at the city level, further verification at the provincial capital level was conducted. The results still showed no significant correlation (Table 11).

**Table 3:** Mediator effect test

Variability	(1)	(2)	(3)	(4)	(5)	(6)	(7)	(8)
	infrastructure construction		Clean energy structure		upgrading of an industrial structure		The Porter Effect	
Clean heating points	0.0144*	0.0261**	0.0566***	0.0497*	0.0226***	0.0276***	−0.0197	−0.0019
	(0.0739)	(0.0120)	(0.0202)	(0.0374)	(0.0048)	(0.0065)	(0.0562)	(0.0327
Control changing	No control	control	No control	control	No control	control	No control	control
City fixed effects	control	control	control	control	control	control	control	control
Time fixed effects	control	control	control	control	control	control	control	control
Observations	1,397	1,397	1,397	1,397	1,397	1,397	1,397	1,397
Adjust	0.974	0.981	0.780	0.883	0.925	0.937	0.914	0.962

### Heterogeneity analysis

The implementation of the clean heating policy directly substitutes scattered coal, while leveraging mechanisms such as the central government’s fiscal multiplier effect, the decarbonization of the energy structure, industrial and sectoral effects, and the transmission of the Porter effect, to reduce regional carbon emission intensity. To evaluate whether the carbon emission reduction effect of the clean heating policy was heterogenous between different cities, the regional heterogeneity was further tested from three aspects: urban administrative level, resource attribute, and presence/absence of coal power output. 

#### Urban administrative level

5. Cities were classified into provincial capitals and nonprovincial capitals based on administrative level to examine whether the carbon reduction effects of clean heating renovations vary across cities of different tiers. The regression results in Table 4 present that the carbon emission reduction effect of the clean heating policy on noncapital cities was significantly negative at the level of 1%, but the effect in capital cities was insignificant. Based on the implementation research of the clean heating policy, provincial capital cities had already conducted a series of environmental governance measures, such as the retrofit of coal-fired boilers, prior to the establishment of clean heating pilot programs. Consequently, the share of scattered coal burning in these cities was low. For example, Beijing took the initiative in launching the “air cleaning action by coal reduction and replacement” in rural areas throughout China since 2013. In noncapital cities, the rural area accounted for a much higher proportion than that in capital cities, and large-scale scattered coal combustion and small coal stove heating, and clean heating renovation were initially implemented mainly after the clean heating pilot policy, so the effect was quite significant after the implementation.

#### Urban resource attributes

6. In 2013, the State Council clearly defined resource-based cities for the first time in the *National Sustainable Development Plan for Resource-based Cities* (2013–2020) and published a list of 262 resource-based cities, of which coal-based cities accounted for about 1/3. Whether a city belonged to a coal resource-based city was distinguished to test regional heterogeneity further by referring to the judgement standard proposed by Yang X M (2014) for coal resource-based cities. The regression results in Table 5 reveal that the clean heating policy exerted a significant carbon emission reduction effect on coal and noncoal resource-based cities, but the correlation coefficient for coal resource-based cities was greater than that for noncoal resource-based cities. This outcome is possibly because the coal mining industry in coal resource-based cities was developed, obtaining scattered coal was convenient for residents, with a long history of scattered coal combustion for heating, so the base of scattered coal combustion was large, and the implementation effect of the clean heating policy was stronger than that in noncoal resource-based cities. Moreover, taking Shanxi Province for example, the accessibility of residents to scattered coal in coal enrichment regions was quite high during the clean heating renovation and local governments even supervised residents to adopt clean heating methods through specific administrative measures. This phenomenon also reinforced the conclusion that the carbon emission reduction effect of the clean heating policy is more significant in coal resource-based cities.

#### Urban coal power output

7. Whether a city belonged to a province with coal power output was distinguished, and the influence of coal power output on the regional carbon emission was explored. Statistical data show that the main provinces with coal power output include Inner Mongolia, Shandong, Shanxi, Shaanxi, and Hebei. The regional heterogeneity was further evaluated on this basis. The regression results in Table 6 reveal that the clean heating policy weakly increased the carbon emission intensity in provinces with coal power output, but it significantly reduced the carbon emission intensity in provinces without coal power output. This outcome is possibly because the large-scale “coal to electricity” transformation increased the power production in provinces with coal power output and increased their carbon emission intensity; in provinces without coal power output, power input or other clean power generation played a dominant role, and clean heating still significantly reduced their carbon emission intensity. Therefore, the clean heating transformation may have caused some carbon transfer between regions.

**Table 4:** Heterogeneity test results of urban administrative hierarchy

variability	Provincial capital		Non-provincial capitals	
	CIgdp1	CIgdp2	CIgdp1	CIgdp2
Clean heating points	−0.0032	−0.0660	−0.1084***	−0.0278***
	(0.0112)	(0.0592)	(0.0086)	(0.0360)
Control changing	control	control	control	control
City fixed effects	control	control	control	control
Time fixed effects	control	control	control	control
Observations	165	165	1,232	1,232
Adjust	0.963	0.991	0.833	0.976

**Table 5:** Heterogeneity test results of urban resource attributes

Variability	Coal resource city		Non-coal resource city	
	CIgdp1	CIgdp2	CIgdp1	CIgdp2
Clean heating points	−0.0401**	−0.1075**	−0.0201**	−0.1105***
	(0.0172)	(0.0618)	(0.0065)	(0.0391)
Control changing	control	control	control	control
City fixed effects	control	control	control	control
Time fixed effects	control	control	control	control
Observations	363	363	1,034	1,034
Adjust	0.867	0.982	0.837	0.837

**Table 6:** Heterogeneity test results of urban coal power output

Variability	Cities that export coal power		Cities that do not export coal power l	
	CIgdp1a	CIgdp1b	CIgdp1a	CIgdp1b
Clean heating points	0.0369*	0.0183**	−0.1494***	−0.1011***
	(0.0216)	(0.0223)	(0.0236)	(0.0343)
Control changing	control	control	control	control
City fixed effects	control	control	control	control
Time fixed effects	control	control	control	control
Observations	57	57	1340	1340
Adjust	0.875	0.903	0.973	0.976

## Discussion

### Effectiveness and limitations of carbon reduction

The empirical analysis reveals the dual nature of the clean heating policy in carbon reduction, it demonstrates clear effectiveness while also exhibiting notable limitations.

First, the carbon reduction effectiveness of the policy has been confirmed through multiple avenues. The Benchmark regression and a series of robustness checks consistently show that the clean heating pilot policy significantly reduces carbon intensity per unit of GDP and per capita carbon emissions in northern regions. This core conclusion remains robust across different carbon emission datasets and when alternative dependent variables are used, confirming the positive impact of the policy on improving “carbon productivity.” The effectiveness is rooted in a clear transmission mechanism: the policy leverages central government funding to stimulate local infrastructure investment, effectively promoting the greening of end-use energy structures and upgrading regional industrial structures. These mechanisms work together to convert policy pressure into tangible structural emission reductions. Additionally, the policy’s effectiveness exhibits clear spatial heterogeneity: in non-provincial capital cities and coal resource-based cities with a high baseline of scattered coal use and large space for transformation, the emission reduction effect is particularly pronounced. This suggests that the allocation of policy resources in specific regions has generated higher emission reduction efficiency.

Secondly, the carbon reduction effect of the policy is fundamentally limited. The most critical finding is that the policy has not produced a statistically significant suppressive effect on total regional carbon emissions. This phenomenon of “intensity reduction without a decrease in total emissions” highlights the limitations of a single terminal consumption-side policy when faced with macro-level emission reduction goals. A more profound limitation is seen in terms of regional equity. The heterogeneity analysis reveals a potential risk: the policy may have increased local carbon intensity in coal-exporting regions. This strongly suggests the possibility of “carbon leakage”, where, to meet the additional electricity demand from the “coal-to-electricity” initiative, coal-rich areas have expanded high-carbon electricity production, causing emissions reductions at the consumption side to come at the cost of increased emissions at the production side. This finding indicates that, without complementary cross-regional carbon accounting and compensation mechanisms, the clean heating policy may improve the local environment while exacerbating carbon inequality between regions.

Additionally, the above conclusions contrast with the findings of Li et al. (2023) [50], whose study concluded that the clean heating policy can reduce total regional carbon emissions. Li et al. used the “regional total energy consumption” and “carbon emission factor” to calculate the total carbon emission. However, the energy consumption of residential life accounts for a small proportion of the regional energy consumption, accompanied by great interregional differences. As displayed in *China Statistical Yearbook 2024* and *Shanxi Statistical Yearbook 2024*, the proportions were 4.05% and 8.02%, respectively, which may contain great deviations. In this paper, a cross-validation of carbon emission data was performed using CEADs and EDGAR, followed by the inversion of the carbon emission data of prefecture-level cities through nighttime light data to over the deviation between calculated data and actual data.

### Paradox of intensity and total emission reduction

The core empirical finding of this paper is the paradox of “intensity reduction without a decrease in total emissions” resulting from the clean heating policy, which reveals a phenomenon that is of universal significance in the early stages of energy transition. This is not a case of policy “failure”, but rather the inevitable result of the competition between “structural effects” and “scale effects” at a specific stage of development. From the perspective of the intrinsic laws of energy-economic system evolution, changes in total carbon emissions can be decomposed into the combined net value of “structural effects” and “scale effects”. The “structural effects” is embodied in the policy’s promotion of cleaner and more efficient end-use energy consumption through technological substitutions such as “coal-to-electricity” and “coal-to-gas”, which directly contribute to the reduction of carbon intensity. This is the key driving force behind the significant reduction in carbon emissions intensity, with the results of the mechanism tests providing strong evidence for this. However, the “scale effects” driven by continued energy consumption is even more pronounced. During the study period (2012–2023), northern China remained in the deepening stages of industrialization and urbanization. The rigid growth in energy consumption due to economic expansion was overwhelming [51]. When the emissions reductions brought about by structural optimization are offset or covered by the new emissions generated by economic scale expansion, this manifests as the macro-level statistical result of “intensity reduction without a decrease in total emissions”. This dynamic competition clearly supports the “decoupling theory” in environmental economics. The findings of this paper suggest that the clean heating policy has effectively promoted the “relative decoupling” of carbon emissions from economic growth in northern regions (where carbon emissions grow at a slower rate than the economy, leading to a reduction in intensity), which is an indispensable intermediate stage towards achieving “absolute decoupling” (where total carbon emissions peak and decline). However, the influence of a single consumption-side policy is insufficient to fundamentally reverse the energy demand growth trend driven by investment, industry, and infrastructure.

From the perspective of econometrics, the DID estimation measures the average treatment effect of the treatment group (pilot cities) relative to the control group (non-pilot cities). The findings of this paper also reveal the structural information behind the DID model results: Firstly, the limited scope of the policy’s impact. The target of the clean heating policy is primarily the heating energy consumption in the residential sector. Although the policy has a broad impact, the share of residential energy consumption in total regional energy consumption and carbon emissions is relatively small (typically around 10%), and its growth rate is often lower than that of high-energy consumption sectors like industry and construction. Therefore, even if the policy produces significant emission reduction effects in the residential sector, these effects may be “drowned out” by the emissions growth in other economic sectors. The insignificance of the total emission coefficient in Table 2 reflects the fact that, from an econometric perspective, the policy’s macro-level impact on total emissions is not statistically visible. Secondly, the heterogeneity of policy effects based on development stages. Most regions in China have not yet reached the carbon peak, which in itself reflects the structural dominance of “scale effects”. In the pre-peak stage, any single, localized emission reduction policy, unless it is strong enough to fundamentally reverse regional growth patterns and energy structures, is more likely to manifest first as an improvement in intensity, rather than a decrease in total emissions. Our heterogeneity analysis indirectly supports this point: in non-coal electricity-exporting regions, the policy significantly reduces carbon intensity, while in coal-exporting regions, the surge in electricity demand has even led to an increase in local emissions intensity. This highlights the complex impact of regional energy system interconnections on the net effects of the policy. 

In summary, the clean heating policy during the evaluation period is a successful “efficiency-enhancing” policy. It has effectively improved the carbon productivity (the inverse of carbon emissions per unit of GDP) in the pilot regions and injected clear “structural improvement” momentum into regional low-carbon transformation. However, as an intervention primarily targeting the consumption side and the residential sector, it inherently has limitations in its contribution to pushing the macro-level carbon emissions total onto a downward trajectory. This conclusion does not negate the value of policy, but rather accurately positions its phased role in China’s “dual carbon” journey. It is an important driver of achieving “relative decoupling”, but to achieve “absolute decoupling”, it is essential to rely on a systemic revolution in the energy supply side (such as deep decarbonization of the electricity system) and a more comprehensive and robust emission reduction policy framework that covers multiple sectors, including industry, transportation, and construction, working in synergy [52].

### Examination of the transmission mechanism

This study not only evaluates the “net effect” of the clean heating policy but also reveals the complex pathways through which the policy influences carbon emissions through mechanism tests. It both confirms the expected channels and uncovers interesting phenomena that differ from theoretical assumptions and existing literature, offering significant policy implications.

Firstly, the study confirms the core mediating role of fiscal leverage and structural transformation. The results in Table 3 support hypotheses H2 and H3, indicating that the policy effectively promotes carbon reduction through two pathways: “central fiscal funding leverage & local investment response” and “cleaning the energy consumption structure”. This finding resonates with Wang Yu et al. (2024) on the fiscal leverage effect, extending the mechanism chain from macro-level financial flows to specific energy structure transformations [53]. It validates that under China’s current fiscal and governance system, central policy signals and financial support can effectively incentivize local government actions and directly drive the low-carbon transformation of end-use energy consumption.

Secondly, an intriguing finding that warrants further exploration is the robustness of the industrial structure upgrading effect, which proves to be stronger than the energy structure effect. This contrasts with the intuitive notion that energy substitution should be the primary mechanism. A possible theoretical explanation for this is that the clean heating policy is not merely an energy substitution project; it is also a large-scale green infrastructure investment and a process of creating low-carbon service markets. The policy implementation has spurred a series of emerging economic activities, ranging from equipment manufacturing, installation and operation to energy efficiency management.

Finally, the failure to validate H4 carries profound policy implications. In contrast to Han et al. who found that technological innovation plays a mediating role in pollution reduction studies [54]. This research suggests that, in the carbon reduction dimension, the policy in its current stage primarily acts as a promoter of mature low-carbon technologies, rather than as a stimulator of breakthrough green innovations. This can be attributed to two main reasons:①The technologies promoted by the policy, such as “coal-to-electricity” and “coal-to-gas”,are relatively mature, and their emission reductions rely more on large-scale application than on cutting-edge breakthroughs [55].②The pressure from policy assessments and renovation timelines encourages local governments and enterprises to prioritize ready-made, reliable solutions rather than investing in long-cycle, high-risk research and development. This distinction carries key policy implications: it suggests that terminal substitution policies designed to achieve rapid large-scale emission reductions may be more innovative in terms of adaptive innovation and business model innovation rather than breakthrough technological innovations measured by patents. Future environmental policy design needs to clearly define its primary goal, whether it is rapid application or innovation inducement, so that the appropriate tools and evaluation frameworks can be matched accordingly.

## Conclusions and suggestions

### Research conclusions

Based on the quasi-natural experiment on the clean heating pilot policy and a theoretical analysis, the panel data of 15 provincial prefecture-level cities in Northern China during 2013–2022 were combined with such methods as multistage DID to test empirically the influence of the clean heating policy on the regional carbon emissions. The potential multiple mediating effects and heterogeneity were discussed. Finally, the following conclusions were mainly drawn:

8. The implementation of the clean heating policy has effectively promoted the decline of carbon emission intensities per unit GPD and per capita in Northern China, which is of great significance for deciding whether to promote clean heating and scattered coal substitution in Northern China continuously. However, this policy achieves no evident effect on the regional carbon emissions, which is possibly attributed to the failure to realize carbon peak in most regions of China.
9. The clean heating policy promotes the regional low-carbon transformation mainly by stimulating local government capital investment, establishing a clean energy structure, and practicing industrial structure upgrading, thus further reducing the carbon emission intensity.
10. Significant heterogeneity is observed in the influence of the clean heating policy on regional carbon emissions in cities at different administrative levels, cities with different resource attributes, and regions with and without coal power output. The policy implementation has a more significant carbon emission impact in noncapital cities, coal resource-based cities, and regions without coal power output, but it may evidently increase the carbon emissions in regions with coal power output.

### Policy suggestions

Based on the research conclusions, the following policy suggestions for implementing and optimizing the clean heating policy are proposed:

First, it is essential to steadfastly advance the clean heating policy by optimizing its implementation across demand, supply, and industrial structure. On the demand side, continued promotion of residential electrification, coupled with optimized subsidies and behavioral guidance, is crucial to prevent coal resurgence. On the supply side, enhancing the capacity for renewable energy generation and grid integration is fundamental for source-side emission reduction. Concurrently, fostering the clean heating service market and intelligent management will unlock further potential. Implementation must be tailored to local resource endowments and development stages to ensure economic sustainability and establish a long-term mechanism.

Second, it is necessary to strengthen the key transmission mechanisms of the policy to unlock synergistic benefits. The guidance and performance supervision of central fiscal funds must be optimized to ensure they effectively leverage local low-carbon investments. Substantial support for the large-scale development and grid integration of renewable energy is required to increase the clean energy share in the supply structure. Furthermore, industrial policies and standards should be leveraged to guide the green upgrading of the related industrial chains, encouraging corporate R&D and innovation to improve energy efficiency and reduce indirect emissions across the value chain.

Third, it is important to implement regionally differentiated policies based on heterogeneous local conditions. Increased resource support should be provided to non-provincial capital cities, where the policy effect is more pronounced, to accelerate retrofitting. Provincial capital cities should develop refined models aligned with their specific urban functions. A critical focus must be addressing the “hidden carbon transfer” in coal power-exporting regions by establishing an inter-regional compensation mechanism based on electricity carbon footprint tracking. This approach, utilizing data from power trading platforms, can ensure national net emission reductions while safeguarding the equitable development interests of all regions.

### Research limitations and expectations

This study focuses on the impact of clean heating on regional carbon emissions but does not delve into the effects of transformation technologies and household-level carbon emissions. To address this limitation, we plan to further investigate micro-level data in subsequent research, focusing on households and different clean heating technology pathways, and evaluate the carbon reduction effects more accurately from the perspective of the carbon footprint.

## Appendix

Figure 5 and Tables 7, 8, 9, 10 and 11 place here.

**Table 7:** Pilot cities of clean heating policy across three pilot batches

Pilot year	Pilot cities
2017	Tianjin, Shijiazhuang, Tangshan, Baoding, Langfang, Hengshui, Taiyuan, Jinan, Zhengzhou, Kaifeng, Hebi, and Xinxiang
2018	Hebei Province: Handan, Xingtai, Zhangjiakou, Cangzhou; Shanxi Province: Yangquan, Changzhi, Jincheng, Jinzhong, Yuncheng, Linfen, Lüliang; Shandong Province: Zibo, Jining, Binzhou, Dezhou, Liaocheng, Heze; Henan Province: Luoyang, Anyang, Jiaozuo, Puyang; Shaanxi Province: Xi’an, and Xianyang
2019	Dingzhou City, Hebei Province*, Xinji City, Hebei Province*, Sanmenxia City, Henan Province*, Jiyuan City, Henan Province*, Tongchuan City, Shaanxi Province*, Weinan City, Shaanxi Province*, Baoji City, Shaanxi Province*, Yangling Demonstration Zone, Shaanxi Province*

Note: * The cities marked are county-level cities or demonstration areas. Due to incomplete data statistics, they are counted as prefecture-level cities

**Table 8:** Regression results with replaced explained variable

Variability	CIpop1		CIpop2	
	(1a)	(1b)	(2a)	(2b)
Clean heating points	−0.0197**	−0.0109**	−0.0841***	−0.0303***
	(0.0116)	(0.0046)	(0.0122)	(0.0100)
Observations	1,397	1,397	1,397	1,397
City fixed effects	control	control	control	control
Time fixed effects	control	control	control	control
Control changing	no	yes	no	yes
Adjust	0.831	0.884	0.993	0.996

**Table 9:** Sample selection bias analysis results

Variability	CIgdp1		CIgdp2	
	Excluding provincial capitals	Excluding the “2 + 26” cities	Excluding provincial capitals	Excluding the “2 + 26” cities
Clean heating points	−0.0278*** (0.0086)	−0.0338*** (0.0092)	−0.1084***(0.0360)	0.1496***(0.0366)
Control changing	control	control	control	control
City fixed effects	control	control	control	control
Time fixed effects	control	control	control	control
Observations	1,232	1089	1,232	1089
Adjust	0.834	0.835	0.976	0.957

**Table 10:** Policy exclusion analysis results of “low-carbon city”

Variability	CIgdp1	CIgdp2
Clean heating points	−0.0266***	−0.1056***
	(0.0077)	(0.0348)
City fixed effects	control	control
Time fixed effects	control	control
Control changing	control	control
Observations	1,397	1397
Adjust	0.863	0977

**Table 11:** Porter effect test of provincial capital cities

Variability	The Porter effect	
	(1)	(2)
Clean heating points	−0.3436*	−0.0216
	(0.1367)	(0.0755)
Control changing	No control	control
City fixed effects	control	control
Time fixed effects	control	control
Observations	165	165
Adjust	0.906	0.982

## Data Availability

No datasets were generated or analysed during the current study.
